# Treatment patterns and clinical characteristics prior to initiating depot typical antipsychotics for nonadherent schizophrenia patients

**DOI:** 10.1186/1471-244X-9-46

**Published:** 2009-07-29

**Authors:** Haya Ascher-Svanum, Xiaomei Peng, Douglas Faries, William Montgomery, Peter M Haddad

**Affiliations:** 1 Eli Lilly and Company, Indianapolis, IN, USA; 2 Lilly USA, LLC, Indianapolis, IN, USA; 3 Eli Lilly Australia Pty Ltd, Macquarie Park, Australia; 4 Greater Manchester West Mental Health NHS Foundation Trust, Manchester, UK; 5 Neuroscience and Psychiatry Unit, School of Psychiatry and Behavioural Sciences, University of Manchester, Manchester, UK

## Abstract

**Background:**

Nonadherence with antipsychotic medication is an important clinical and economic problem in the treatment of schizophrenia. This study identified treatment patterns and clinical characteristics that immediately precede the initiation of depot typical antipsychotics in the usual treatment of schizophrenia patients with a recent history of nonadherence with oral antipsychotic regimens.

**Methods:**

Data were drawn from a large, multisite, 3-year prospective noninterventional observational study of persons treated for schizophrenia in the United States, which was conducted between 7/1997 and 9/2003. The analytical sample included patients who, in the 6 months prior to enrollment, were considered nonadherent with oral antipsychotics and were not treated with depot antipsychotics (N = 314). Patients who were subsequently initiated on typical depots during the 3-year follow-up were compared with patients who continued therapy with only oral antipsychotic agents. Group comparisons were made on patient baseline characteristics and precedent variables that were assessed 1 to 6 months prior to depot initiation. Patient assessments were made at predetermined intervals throughout the 3-year study using standard psychiatric measures, a patient-reported questionnaire, and medical record information.

**Results:**

A small proportion of patients (12.4%) who were recently nonadherent with oral antipsychotics were subsequently initiated on depot therapy during the 3-year study. Compared to patients treated with only oral antipsychotics, those subsequently initiated on a depot were significantly more likely to be hospitalized at depot initiation or the previous 30 days, to have recent involvement with the criminal justice system (arrests), recent illicit drug use, recent switching or augmentation of oral antipsychotics, and recent treatment with oral typical antipsychotics.

**Conclusion:**

Despite prior nonadherence with oral antipsychotic medication, only a small proportion of nonadherent schizophrenia patients were initiated on depot antipsychotics in this 3-year prospective study. Patients subsequently initiated on depot had a more severe treatment pattern and clinical profile immediately preceding depot initiation. This profile may have triggered the decision to initiate a depot. Findings have important clinical and economic ramifications for practitioners, policy makers, and other decision makers, highlighting the need for early identification of and tailored therapeutics for schizophrenia patients with a history of nonadherence with their recent oral antipsychotic regimens.

## Background

Schizophrenia is a severe and chronic mental illness that requires long-term, often lifetime, treatment with antipsychotic medications [1]. The use of these agents has been shown to reduce the risk of relapse and hospitalization and help improve patients' long-term functional outcomes [2-4]. Despite this, nonadherence is highly prevalent among schizophrenia patients, with at least one-third estimated to be nonadherent with their medication regimens [5].

Compared to adherence, nonadherence with antipsychotic medication has been shown to be a robust predictor of relapse, hospitalizations, and poorer long-term functional outcomes, including greater likelihood of being arrested, of being violent, and of becoming a victim of violent crimes [6-9].

Given the pervasive problems associated with nonadherence to oral antipsychotic regimens, long-acting injectable formulations (depots) have been developed. Available depot antipsychotics include typical antipsychotics (e.g., haloperidol and fluphenazine decanoate) and 2 atypical antipsychotics (risperidone long-acting injection and olanzapine long-acting injection [FDA approval pending in the United States]). Depot antipsychotics are recognized as a safe and effective strategy for improving medication adherence [10,11] and appear to benefit nonadherent schizophrenia patients [12]. While the use of depot for the treatment of first-episode psychosis is often debated, most treatment guidelines recommend that depots be considered in the management of nonadherent patients [1,13-15].

Although depot antipsychotics are generally targeted for nonadherent patients, only a small proportion of schizophrenia patients are treated with depots in the United States [16]. To improve understanding of this phenomenon, previous U.S.-based research assessed the characteristics of schizophrenia patients who are treated with depot compared to those who are not [16-18]. Previous studies have shown depot recipients to differ from non-depot recipients on several characteristics. For example, a recent U.S. study has found that patients treated with depot typicals were more likely to be African-American, to have had involvement with the criminal justice system, to use alcohol or illicit substances, and to have psychiatric hospitalizations [18]. However, time-sensitive variables were not necessarily assessed immediately prior to depot initiation due to the study methodology.

Although certain individuals may be more likely to receive depot antipsychotics than others, it is unclear whether there are specific treatment patterns and clinical characteristics that immediately precede and thus may "trigger" the initiation of depot antipsychotics for schizophrenia patients who are deemed nonadherent with their oral antipsychotics. To this end, the present study used data from a large U.S. multisite, prospective, 3-year noninterventional observational study of persons treated for schizophrenia in the United States (US-SCAP) [19-21] to assess treatment patterns and clinical characteristics that immediately precede (by 1 to 6 months) the initiation of depot typical antipsychotics in the treatment of patients who were recently nonadherent to their oral antipsychotic regimens.

## Methods

### Data Source

We used data from the U.S. Schizophrenia Care and Assessment Program (US-SCAP), a large, naturalistic, prospective, multisite, noninterventional study in which patients treated for schizophrenia-spectrum disorders were periodically assessed and followed for 3 years. US-SCAP was conducted between July 1997 and September 2003; the goal of the study was to understand the treatment of schizophrenia patients in usual-care settings. Patients were recruited from urban and rural areas and from diverse geographical regions, including the Northeast, Southwest, Mid-Atlantic, and West. The 6 participating regional sites represented large systems of care, including community mental health centers, university health care systems, community and state hospitals, and the Department of Veterans Affairs Health Services. Institutional Review Board approval was obtained and informed consent was received from all patients. US-SCAP findings have been published elsewhere [18-21].

US-SCAP participants underwent clinical assessments with standard psychiatric measures at baseline and 12-month intervals thereafter (e.g., the Positive and Negative Syndrome Scale [PANSS]) [22]. Patient-reported assessments using the SCAP-Health Questionnaire (SCAP-HQ) [23] were administered at baseline and 6-month intervals thereafter. In addition, systematic abstraction of patients' medical records occurred at 6-month intervals by trained and certified examiners, using a medical record abstraction form developed for this study. The medical records provided medication prescription information and data on use of psychiatric services (e.g., hospitalization).

### Sample

The analytical sample for this study included US-SCAP participants who, during the 6 months prior to enrollment, were deemed nonadherent with their oral antipsychotics and were not treated with depot antipsychotics (recently nonadherent) at entry into the study or in the 6 months prior to enrollment. These participants also had to have at least 1-year follow-up in the study to enable identification of patients who were subsequently initiated on depot. The recently nonadherent patients were assigned to 2 mutually exclusive groups: 1) *depot initiators*, those who were initiated on any typical depot antipsychotic at any time during the 3-year follow-up, or 2) *non-depot initiators*, those who were not initiated on typical depot antipsychotic during the following 3 years.

### Definition of Adherence

Adherence was assessed using both medical record medication information and patient-reported adherence. Medical record medication information for each antipsychotic medication prescribed to the patient (e.g., medication name, formulation, dosage, starting and stopping dates) was used to calculate a Medication Possession Ratio (MPR) for each patient. MPR is a common proxy measure of medication adherence and is typically used in claims database analyses [6,7,16,19,20]. For this study, MPR was calculated as the percentage of total days with any oral antipsychotic medication in the 6 months prior to enrollment. Consistent with prior research [8], MPR ≥ 85% was defined as being adherent and MPR <85% was defined as nonadherent.

Patient-reported medication adherence was assessed at baseline and reflected reported adherence with medication in the previous 4 weeks. The adherence item on the SCAP-HQ, a health questionnaire developed and validated for the SCAP study [23], was used. This item is rated on a 5-point scale: 1) I never missed taking my medicine; 2) I missed only a couple of times, but basically took all the medicine; 3) I missed the medicine several times, but took at least half of it; 4) I took less than half of what was prescribed; and 5) I stopped taking the medicine altogether. Participants who chose alternative 1 or 2 were considered adherent per self report while all others were classified as "poorly adherent" [20].

The medical record-based MPR and the patient-reported adherence were then used to define medication adherence in the 6 months prior to enrollment. Participants who reported being adherent at enrollment and had an MPR ≥ 85% during the 6 months prior to enrollment were defined as adherent. All others were deemed nonadherent.

### Baseline Variables

Variables gathered at baseline (enrollment) included age, gender, ethnicity, marital status, education level, health insurance, PANSS total score, positive, negative, and general psychopathology scores, age at illness onset, comorbid mental retardation or borderline intelligence, and supervised housing arrangements.

### Precedent Variables

Post-baseline variables that immediately preceded depot initiation or noninitiation included treatment pattern in the prior 90 days: use of any atypical antipsychotic, any typical antipsychotic, any antidepressant, any oral antipsychotic or antidepressant, and switching or augmentation (with another antipsychotic) of oral antipsychotics. In addition, the following utilization of acute psychiatric services was assessed: at least 1 psychiatric hospitalization in the 6 months prior to depot initiation, hospitalization for psychiatric purposes at depot initiation or the 30 days prior to initiation, multiple (2 or more) psychiatric hospitalizations between enrollment and depot initiation, and prior use (past 4 weeks) of emergency psychiatric services. Finally, clinical characteristics assessed with the SCAP-HQ included substance use (alcohol use and illicit drug use in past 4 weeks), being arrested or jailed (in the past 6 months), being violent, being victimized by others, having suicidal thoughts, making suicidal threats, and making a suicide attempt (in the past 4 weeks).

### Data Analysis

Baseline (enrollment) characteristics of recently nonadherent depot initiators (N = 39) were compared with recently nonadherent non-depot initiators (N = 275) using the Fisher's exact test for categorical variables and t-tests for continuous variables.

Precedent variables from depot initiators were compared with non-depot initiators as follows: Treatment patterns and clinical characteristics that preceded initiation of depot were taken from the data collection point closest to the date of depot initiation for the depot-initiators group and compared with the data collection point closest to day 269 post-enrollment for the non-depot initiator group. This date was utilized for the non-depot initiating group to provide a control group, as this was the mean time from enrollment to depot initiation for depot initiators. Fisher's exact test and t-tests were utilized for the group comparisons. The differences in precedent variables between depot and non-depot patients were also assessed using a propensity score-adjusted bootstrapping method. This provided for an analysis of group differences adjusted for differences in baseline characteristics to complement the unadjusted analysis above. The variables used for adjusting were: gender, race, high school education (Y or N), typical antipsychotic medication use in the 90 days prior to depot initiation (Y or N), antidepressants use in the 90 days prior to depot-initiation (Y or N), substance use in the 6 months before enrollment (Y or N), legal problems in the 6 months before enrollment (Y or N), and psychiatric hospitalization in the 30 days before depot initiation (Y or N). To assess robustness of the findings, we conducted a sensitivity analysis, using propensity scores to match the time point of each non-depot initiator to that of each depot initiator with a similar score. The precedent variables were then compared at matched time points between depot and non-depot initiators. We also conducted a second sensitivity analysis in which the control group was chosen on the basis of a change in medication. This was done because the initiation of depot treatment is a decisional process reflecting a need to change the current medication regimen. The new control group was comprised of patients who were previously nonadherent with their oral antipsychotic, have not been initiated on depot but have undergone a medication switch to another oral antipsychotic, or were augmented with another oral antipsychotic.

## Results

### Depot Initiation

Only a small proportion of the 314 patients (n = 39 or 12.4%) who were previously nonadherent with oral antipsychotics was subsequently initiated on depot therapy during the 3-year study. Most depot initiations occurred in the first year following patients' enrollment in the study.

### Baseline Variables

Table 1 presents the baseline (enrollment) variables for patients who were subsequently initiated on depot and patients who were not. The only significant difference between the 2 groups was age at baseline, with younger individuals more likely to receive depot antipsychotics.

**Table 1:** Baseline characteristics of recently nonadherent patients who were subsequently initiated on depot and patients who were not

Variable	Depot Initiators (n = 39)	Non-Depot Initiators (n = 275)	Comparison P-value
Age at baseline, mean (SD)	34.7 (10.8)	40.8 (11.0)	.001
Gender, Male, n (%)	25 (64.1%)	157 (57.1%)	.489
Race, n (%)			.339
White	17 (43.6%)	153 (55.8%)	
Black	19 (48.7%)	102 (37.2%)	
Other	3 (7.7%)	19 (6.9%)	
Marital status, married, n (%)	13 (33.3%)	122 (44.9%)	.226
High school education or higher, n (%)	21 (53.8%)	182 (66.7%)	.150
Age at illness onset, mean (SD), years	17.7 (6.5)	19.2 (9.5)	.373
Health Insurance, n (%)			.771
Medicaid	29 (74.4%)	180 (65.5%)	
Medicare	11 (28.2%)	95 (34.5%)	
Medicaid and Medicare	8 (20.5%)	54 (19.6%)	
PANSS total score at enrollment, mean (SD)	70.3 (20.6)	68.8 (18.2)	.711
PANSS positive score at enrollment, mean (SD)	16.9 (6.6)	16.0 (6.1)	.516
PANSS negative score at enrollment, mean (SD)	18.8 (6.2)	17.5 (5.5)	.291
PANSS general psychopathology score at enrollment, mean (SD)	35.4 (11.4)	35.3 (10.0)	.970
Mental retardation/borderline IQ, n (%)	2 (5.1%)	5 (1.8%)	.211
Supervised housing, n (%)	4 (10.3%)	19 (6.9%)	.506

### Precedent Variables

Table 2 presents results of the comparisons between depot initiators and non-depot initiators on prior treatment patterns and clinical characteristics immediately preceding depot initiation. Compared to patients not initiated on depot, those who were depot initiators were more than twice as likely to have been treated with oral typical antipsychotics in the prior 90 days, were significantly less likely to use antidepressants, and were about 3 times more likely to have undergone recent (previous 90 days) switching or augmentation of their oral antipsychotics. Depot initiators were also significantly more likely to have had at least 1 recent psychiatric hospitalization (in the previous 6 months), to have been hospitalized at initiation or the 30 days prior to depot initiation, to have had multiple (at least 2) psychiatric hospitalizations between study enrollment and depot initiation, and to have had a shorter time (measured in days) to first hospitalization after enrollment in the study. Overall, the depot initiators were about 8 times more likely to be hospitalized at initiation or the 30 days prior to depot initiation compared to non-depot initiators. Depot initiators were also significantly more likely to report recent illicit drug use (in the previous 4 weeks) and to have been recently arrested or jailed (in the previous 6 months). The 2 patient groups did not significantly differ on recent use of oral atypical antipsychotics (in the previous 90 days), on recent use of emergency psychiatric services (in the previous 4 weeks), alcohol consumption, violent behavior, being a victim of crime, or having suicidal thoughts, making suicide threats, and attempting suicide. Compared to non-depot initiators, the depot initiators were significantly more likely to have recently experienced both a psychiatric hospitalization and legal difficulties (0.4% vs. 23.1%, p < .001) and both psychiatric hospitalization and illicit drug use (0.7% vs. 7.7%, p = .015). The groups did not significantly differ on the proportion of patients with both recent legal difficulties and recent drug use or on the proportion of patients with recent psychiatric hospitalization, legal difficulties, and drug use.

**Table 2:** Comparisons of depot initiators and non-depot initiators on prior treatment patterns and clinical characteristics immediately preceding depot initiation

Variable	Depot Initiators (n = 39)	Non-Depot Initiators (n = 275)	Comparison P-Value
**Prior treatment pattern**, * n (%)			
Use of oral typical antipsychotics	26 (66.7%)	69 (25.1%)	<.001
Use of oral atypical antipsychotics	19 (48.7%)	164 (59.6%)	.226
Use of antidepressants	11 (28.2%)	128 (46.5%)	.038
Switch/augment oral antipsychotics	17 (43.6%)	37 (13.5%)	<.001
**Prior acute psychiatric services**, n (%)			
Hospitalized in 6 months prior to depot initiation	25 (64.1%)	41 (14.9%)	<.001
Multiple (≥ 2) hospitalizations between enrollment and depot initiation	14 (35.9%)	39 (14.2%)	.001
Hospitalized at or 30 days prior to depot initiation	22 (56.4%)	18 (6.5%)	<.001
Time to first hospitalization post-enrollment, mean (SD)	168.9 (140.8)	373.7 (332.5)	<.001
Emergency psychiatric services**	3 (7.7%)	25 (9.1%)	.999
**Prior clinical variables**, n (%)			
Recent alcohol use**	12 (30.8%)	59 (21.5%)	.220
Recent illicit drug use**	8 (20.5%)	24 (8.7%)	.041
Recent alcohol or illicit drug use**	14 (35.9%)	66 (24.0%)	.119
Violent behavior**	3 (7.7%)	24 (8,7%)	.999
Victim of crime**	6 (16.2%)	36 (13.1%)	.608
Arrested/jailed ***	9 (23.1%)	18 (6.5%)	.002
Suicidal thought or threat**	4 (10.3%)	43 (15.6%)	.478
Suicide attempt**	0 (0)	10 (3.6%)	.619

*During the 3 months prior to depot initiation (for initiators) or prior to the mean time to depot initiation (for non-depot initiators)

**During the 4 weeks prior to the assessment closest to the date of depot initiation (for initiators) or prior to the mean time to depot initiation (for non-depot initiators)

***During the 6 months prior to the assessment closest to the date of depot initiation (for initiators) or prior to the mean time to depot initiation (for non-depot initiators)

### Sensitivity Analyses

Results of the first sensitivity analysis, in which propensity scores were used to match the time point of each non-depot initiator to that of a depot initiator with a similar score, were essentially unchanged. For example, depot initiators were twice as likely to have been treated with oral typical antipsychotics in the prior 90 days (66.7% vs. 26.6%), were significantly more likely to have had at least 1 recent psychiatric hospitalization (in the previous 6 months, 64.1% vs. 19%), to have been hospitalized at initiation or the 30 days prior to depot initiation (56.4% vs. 9.6%), and to have had a shorter time (measured in days) to first hospitalization after enrollment in the study (168.9 days vs. 366.9 days).

Results of the second sensitivity analysis were also essentially unchanged. This analysis used another control group, which comprised nonadherent patients who were treated with an oral antipsychotic, were not initiated on depot and have undergone a medication switch, or an augmentation with another antipsychotic. This control group was smaller than the original control group (118 instead of 275 patients). As in the original findings, the depot initiators were significantly younger than the control group, were more likely to have been treated with oral typical antipsychotics in the prior 90 days, to have had at least 1 recent psychiatric hospitalization (in the previous 6 months), to have been hospitalized at initiation or the 30 days prior to depot initiation, to have had multiple (at least 2) psychiatric hospitalizations between study enrollment and depot initiation, and to have had a shorter time (measured in days) to first hospitalization after enrollment in the study. Overall, the depot initiators were about 4 times (compared to 8 times in the original analysis) more likely to be hospitalized at initiation or the 30 days prior to depot initiation compared to the non-depot initiators. There were, however, 2 variables that lost their original statistical significance: treatment with antidepressants and recent use of illicit drugs. Although the direction of the findings remained unchanged, the loss of statistical significance was likely due to a smaller sample size.

## Discussion

This study revealed several important findings. First, although consensus guidelines for the treatment of schizophrenia stipulate the choice of depot antipsychotics for nonadherent patients, only a small proportion of nonadherent patients (12.4%) were initiated on depot antipsychotics in this 3-year prospective observational noninterventional study. Second, when focusing on patients with schizophrenia who were recently nonadherent with their oral antipsychotics, several treatment and clinical variables appear to immediately precede the initiation of the depot antipsychotic medication, including use of oral typical antipsychotics, switching or augmentation of oral antipsychotics, a psychiatric hospitalization, prior multiple hospitalizations, illicit drug use, and involvement with the criminal justice system. Lastly, this study found that a recent psychiatric hospitalization may be a major "trigger" of depot initiation, as depot initiators were about 8 times more likely to be hospitalized at the time of depot initiation or in the 30 days immediately prior to initiation compared to non-depot initiators (56% vs. 6.5%). Having both a recent psychiatric hospitalization and recent legal difficulties also emerged as a robust trigger of depot initiation, but this marker was applicable to a smaller proportion (23%) of depot initiators.

These findings suggest there may be a distinct treatment pattern and clinical profile that "triggers" clinicians to initiate depot antipsychotics. Current findings are consistent with previous research suggesting that certain individuals are more likely to receive depot antipsychotic formulations [16,18,24]. Results of those studies have demonstrated that individuals receiving depot antipsychotics were more likely to be African-American and younger than those not receiving depots. While the present results did not demonstrate an effect of race within the recently nonadherent group, they extend previous findings in that other precedent variables were associated with depot therapy. Taken together, it appears that recently nonadherent individuals with a more serious recent clinical and treatment profile (i.e., requiring hospitalization, comorbid illicit substance use, or involvement with the criminal justice system) may be more likely to receive depot antipsychotics.

The current findings also show that depot initiators were more likely to have been recently treated with oral typical antipsychotics and to have undergone recent switching or augmentation of their oral antipsychotic. The drivers of these findings are unclear as the study did not assess reasons for medication initiation or discontinuation. It is possible that both recent use of typical antipsychotics and antipsychotic switching/augmentation reflect suboptimal effectiveness and/or greater medication intolerability in this patient group, thus leading clinicians to alter antipsychotic treatment in an attempt to improve patients' outcomes. We also assessed the possibility that depot initiators were less adherent than non-depot initiators with their antipsychotics during the period just preceding depot initiation. However, this hypothesis was not supported by the data (results not shown). The finding may also reflect reluctance by clinicians to switch patients without recent exposure to oral typical drugs to a typical depot. In some cases, there may be a good reason for this; for example, a patient may have experienced extrapyramidal side effects with typical antipsychotics, leading to a switch to an atypical.

While this study used data of recently nonadherent schizophrenia patients to help identify which patients with what recent clinical characteristics are more likely to be subsequently initiated on depot, a recent survey by West and colleagues [25] of U.S. psychiatrists assessed similar clinical variables that may influence psychiatrists' decisions to prescribe depot antipsychotics. Our results that 12.4% of the nonadherent patients were initiated on depot are strikingly consistent with findings of West and colleagues' study, in which a small proportion of psychiatrists (17.6%) reported initiating depot antipsychotics in nonadherent patients. Moreover, depot initiation was reported by psychiatrists to be more likely for patients who had previously been hospitalized. Taken together, the present results and those reported by West and colleagues suggest that only a small subset of schizophrenia patients appears to receive depot antipsychotics in the United States, possibly because only a small subset of psychiatrists is ready to use depot antipsychotics, despite much higher prevalence of medication nonadherence in this patient population. The study by West and colleagues was conducted shortly after the introduction of risperidone long-acting injection in the United States, suggesting that the availability of the first atypical depot had not had a major impact on clinicians' reluctance to initiate depot treatment. The low use of depots may partly reflect patient-led barriers. For example, some patients may not like injections and others may decline antipsychotic treatment irrespective of whether it is in oral or depot form. In usual practice, the low use of depots is likely to reflect a combination of clinician- and patient-led factors.

The current findings need to be interpreted in the context of this study's limitations. First, due to the low rate of depot initiation, our sample size was small. This adversely impacted the power for statistical testing and the precision in estimation, thus reducing our ability to detect differences in baseline and precedent measures between the patient groups. Nevertheless, differences were large enough that statistically significant effects were found in multiple precedent measures. Small sample size also limited our ability to fully adjust our analysis for baseline group differences. Second, participants in US-SCAP were assessed using the PANSS at 12-month intervals throughout the 3-year study. Because these assessments were not set to coincide with the time of medication initiation or discontinuation, patients' symptom levels were not available at the time of initiation, nor were the reasons for the decision to initiate the medication. Third, the present study was a post hoc analysis that will require further confirmatory research. And lastly, the study did not collect information about patients declining clinicians' recommendations of depot antipsychotics, thus possibly had underestimated the rate with which depots are proposed to these patients as the appropriate formulation choice.

## Conclusion

This study found that even among recently nonadherent schizophrenia patients – the patient segment recommended for depot antipsychotics by treatment guidelines – only a small proportion appears to be treated with depot antipsychotic therapy over a 3-year period. Individuals initiating depot therapy were more likely to be young and have a more severe clinical and treatment profile immediately prior to depot initiation. Future research is needed to prospectively replicate these findings and determine which other variables may influence depot initiation in the long-term treatment of patients with schizophrenia.

## Competing interests

Haya Ascher-Svanum, Xiomei Peng, Douglas Faries, and William Montgomery are full-time employees of Eli Lilly and Company and minor shareholders. Peter Haddad has received fees for lecturing and consultancy from the manufacturers of various antipsychotics including Astra Zeneca, Bristol-Myers Squibb, Eli Lilly, and Janssen-Cilag.

## Authors' contributions

HA-S conceived of the study, helped design the study, participated in developing the analytical plan, and wrote the manuscript. DF prepared the analytical plan and participated in the writing of the manuscript. XP conducted the Statistical analyses. PH and WM helped critically revise the manuscript and draft the manuscript. All authors read and approved the final manuscript.

## Pre-publication history

The pre-publication history for this paper can be accessed here:


